# Glaucoma Management Therapies and Clinical Outcomes in an African Population: A Review of Prospective Studies

**DOI:** 10.3390/jcm15051837

**Published:** 2026-02-28

**Authors:** Albert Kwadjo Amoah Andoh, Kwadwo Owusu Akuffo, Josephine Ampong, Kwadwo Antwi Appiagyei, Isaiah Osei Duah Junior, Simon Christoph König, Alexander Karl-Georg Schuster

**Affiliations:** 1 Department of Optometry and Visual Science, College of Science, Kwame Nkrumah University of Science and Technology, Private Mail Bag, UPO, KNUST, Kumasi 00233, Ghana; andohalbertamoah@gmail.com (A.K.A.A.); josephineampong5@gmail.com (J.A.); mrkappiagyei@gmail.com (K.A.A.); oseiduahisaiah@gmail.com (I.O.D.J.); 2 Department of Biological Sciences, College of Science, Purdue University, West-Lafayette, IN 47907, USA; 3 Department of Psychology, John R. and Kathy R. Hairston College of Health and Human Sciences, North Carolina Agricultural and Technical State University, Greensboro, NC 27411, USA; 4 Centre for Ophthalmic Epidemiology and Healthcare Research, Department of Ophthalmology, University Medical Centre Mainz, 55131 Mainz, Germany; simon.koenig@unimedizin-mainz.de

**Keywords:** glaucoma, treatment, surgery, laser therapy, pharmacological therapy, Africa

## Abstract

Glaucoma is a leading cause of irreversible blindness in Africa, with disease burden exacerbated by limited access to eye care, shortages of trained ophthalmologists, and socioeconomic disparities. This review synthesizes prospective and interventional studies evaluating glaucoma management modalities and associated clinical outcomes in an African population. Trabeculectomy remains the predominant surgical intervention, achieving success rates of approximately 80%, with enhanced outcomes when augmented with anti-metabolites such as mitomycin-C or 5-fluorouracil. Laser-based interventions, including selective laser trabeculoplasty and transscleral cyclophotocoagulation, demonstrate moderate to high efficacy in reducing intraocular pressure, while nonpenetrating surgeries such as deep sclerectomy, viscocanalostomy, and canaloplasty provide substantial pressure reduction with fewer complications. Pediatric interventions, notably goniotomy, show efficacy in lowering intraocular pressure, although region-specific evidence remains limited. Evidence on pharmacological intervention remains scarce, fragmented, with high rates of non-adherence frequently reported, highlighting the need for rigorously-designed outcome-oriented studies to inform clinical practice. Adoption of newer surgical and laser techniques is constrained by cost, limited equipment, and insufficient subspeciality expertise. Improving glaucoma outcomes in Africa will require strategic expansion of access to effective treatments, strengthen local surgical capacity, and prioritize evidence-based research. Collectively, these efforts will provide a more robust framework to optimize glaucoma management and reduce the burden of irreversible blindness across the continent.

## 1. Introduction

Glaucoma is a neurodegenerative eye disease characterized by progressive optic neuropathy, irreversible visual field loss, and/or intraocular pressure (IOP) as a risk factor [1,2]. Its estimates continue to vary by geographic region [3]. Glaucoma affects an estimated 3.54% of the global population aged 40 to 80 years, with primary open-angle glaucoma (POAG) being most prevalent in Africa (4.20%) and primary angle-closure glaucoma (PACG) most common in Asia (1.09%) [4]. The incidence of glaucoma is notably higher in regions with a low sociodemographic index and on the African continent, with pooled annual cumulative incidence estimates of 0.21% for POAG and 0.05% for PACG [5].

Globally, glaucoma management encompasses pharmacological, laser, and surgical interventions [6]. Despite these established treatment modalities, substantial disparities persist in therapeutic outcomes, with reported effect sizes of 0.39 for laser trabeculoplasty and 0.66 for medical management, while surgical interventions demonstrate the highest effectiveness at 0.73 in Sub-Saharan Africa [7]. Notably, comparative studies have identified marked differences in disease progression and treatment responsiveness among African, European, and Asian populations [8,9]. In individuals of African descent, for instance, prostaglandin analogues demonstrate higher IOP-lowering efficacy, whereas beta-adrenergic blockers are generally less effective. Additionally, while laser trabeculoplasty has shown favorable IOP reduction in this group, trabeculectomy failure rates remain disproportionately high [8], reflecting a critical need for population-specific treatment strategies and evidence-based optimization of glaucoma care across diverse ethnic contexts.

Within the African context, robust evidence on glaucoma management outcomes remains limited, with most available studies focusing on glaucoma epidemiology rather than comprehensive assessments of treatment outcomes and complications. Population-based data predominantly focus on glaucoma epidemiology and are confined to Sub-Saharan Africa, leaving significant gaps in understanding the effectiveness and safety of therapeutic options across the continent. Consequently, existing reviews offer only a partial representation of the continental glaucoma burden, lacking an integrated synthesis of treatment modalities, outcomes, and associated complications. There is thus a pressing need for continent-wide analyses that consolidate data on glaucoma management to elucidate regional patterns and therapeutic outcomes in Africa.

To address this need, this review synthesizes evidence-based studies reporting on treatment modalities and outcomes across Africa. A comprehensive search was conducted in PubMed, Scopus, Google Scholar, and African Journals Online for studies published from inception to 31 December 2024. Only published, peer-reviewed articles in the English language were synthesized. Prospective studies on the treatment and management of glaucoma across Africa, focusing on the most rigorous evidence for evaluating therapeutic efficacy, safety, and clinical applicability, were included in this review. Particularly, randomized controlled trials (RCTs) or prospective non-randomized studies assessing treatment outcomes among African participants with confirmed glaucoma, as well as evaluating the effectiveness of therapeutic interventions, including medical (e.g., topical agents), laser-based, and surgical treatments, were included in the review. Primary outcomes focused on IOP reduction, while secondary outcomes included treatment-related complications.

Treatment outcomes were narratively synthesized to examine available treatment options along with their efficacy and complications, and future implications for reducing the burden of glaucoma on the continent. Additionally, the review not only consolidates what is known but also reveals major deficits and underreporting in key areas such as medical therapy outcomes. By highlighting management outcome inconsistencies, this review informs future research priorities and supports regionally tailored strategies for treatment accessibility, affordability, and policy development.

## 2. Treatment Modalities for Management of Glaucoma in Africa

Surgical success was typically defined as IOP < 21 mmHg, >20% IOP reduction without medication, or avoidance of further surgery, with follow-up ranging from 3 to 35 months (see Table 1).

### 2.1. Surgical Therapy

Surgical interventions were the most common treatment modality, with trabeculectomy-based procedures evaluated in most studies, followed by deep sclerectomy, viscocanalostomy, goniotomy, and canaloplasty.

#### 2.1.1. Trabeculectomy

Trabeculectomy outcomes in Black patients have been a subject of research for decades, and studies have reported high success rates of 80% in Black American patients [24], similar to findings in Africa [12]. Two RCTs in Kenya and Congo showed enhanced IOP reduction with trabeculectomy using 5-Fluorouracil (5-FU) [12] and Mitomycin-C (MMC) [10] anti-metabolites. Yorston et al. found 88.8% of 5-FU-treated eyes maintained controlled IOP at two years vs. 70.6% with placebo [12]. Mwanza and Kabasele reported 81.8% success with MMC vs. 63.6% without, and greater IOP reduction (57.9% vs. 42.9%) [10]. In Ghana, 5-FU reduced IOP from 29.2 to 17.3 mmHg with 87% success [11]. Beta radiation is an alternative to anti-metabolites, with lowered failure rates (5% vs. 30%) and improved IOP control at six months [13].

More recently, ab interno trabeculectomy showed comparable efficacy and complication rates between African American and Caucasian patients, with significant IOP reduction in both groups [25]. In a randomized trial, Singh et al. reported superior outcomes with intraoperative MMC, achieving IOP < 21 mmHg in 93.2% of cases versus 73% with 5-FU; for IOP < 15 mmHg, success was 63.6% with MMC and 51.4% with 5-FU [14]. Trabeculectomy augmented with MMC has consistently outperformed both unaugmented trabeculectomy and 5-fluorouracil (5-FU) [10] however, its use is associated with a higher risk of complications, including cataract formation, hypotony, blebitis, superficial keratitis, uveitis, and even vision loss [10,13,14]. In response to these concerns, alternative surgical procedures such as deep sclerectomy, viscocanalostomy, and canaloplasty have been explored. These techniques have shown promising results in selected populations, with success rates exceeding 80% and fewer postoperative complications [21]. Nonetheless, their adoption may be limited by higher procedural costs, particularly in the case of viscocanalostomy and microcatheter-assisted canaloplasty. See Table 2.

#### 2.1.2. Other Surgical Approaches

One prospective study showed deep sclerectomy reduced IOP from 20.2 ± 6.1 mmHg (medicated) and 30.7 ± 9.8 mmHg (unmedicated) to 12.1 ± 4.1 mmHg at 12 month, resulting in a 40.3% and 60.6% reduction, respectively [19]. Stegmann et al. reported 64% IOP reduction with viscocanalostomy in 214 OAG eyes, with complete and qualified success rates of 82.7% and 89% at 35 months [21]. Similarly, canaloplasty in 60 POAG patients achieved 65.8% IOP reduction and success rates of 77.5% (complete) and 81.6% (qualified) at three years [20] (see Table 2). For pediatric glaucoma, this review found one randomized controlled trial in Africa. A prospective randomized study on goniotomy in a pediatric population using a 23-gauge needle demonstrated the greatest postoperative IOP-lowering effect of 8.97 mmHg, followed by the microvitreoretinal blade at 9.55 mmHg and the Kahook blade at 9.77 mmHg [18]. 

Goniotomy is a preferred surgical procedure for managing congenital and infantile glaucoma, demonstrating significant IOP reduction across various techniques [18]. Data on the epidemiology of congenital or juvenile glaucoma remains sparse in Africa, however, goniotomy proves effective in pediatric glaucoma patients, though studies on the procedure remain limited. Among the available evidence, postoperative IOP reduction varied slightly depending on the surgical instrument used [18]. Another way of treatment without the need for a tiltable microscope is performing a trabeculotomy, a trabeculectomy, or a combination of both [26].

### 2.2. Laser Therapy

African individuals have been reported to respond better to laser surgery, while Caucasians patients to trabeculectomy [27]. Selective Laser Trabeculoplasty (SLT) has been reported to offer success rates between 60% and 70% and IOP reductions of up to 27.6% [22,23,28]. Similarly, in the West Indies, SLT monotherapy achieved a 29.7% to 39.5% IOP reduction at 12 months in Afro-Caribbean patients [29]. One comparative RCT of 201 patients compared Timolol 0.5% with SLT, with SLT showing significantly better outcomes, and a 61% success rate (99 patients) compared to 31% for Timolol (55 patients). The odds ratio of 3.37 (*p* < 0.0001) strongly supported SLT’s superiority over Timolol [16].Two prospective studies on SLT also demonstrated strong evidence for reducing IOP with minimal complications, with success rates ranging from 60% [22] to 70% [23] and IOP reductions of 18% [23] to 27.6% [22]. One prospective clinical trial investigating the feasibility of a diode laser transscleral cyclophotocoagulation (TSCPC) as a primary surgical treatment for POAG also reported a decreased IOP in 53 eyes (67%), with a reduction of ≥20% in 37 eyes (47%) and final IOP ≤ 22 mmHg in 38 eyes (48%) [17]. While several studies have reported on the relatively short-term effectiveness of laser treatment in the African population, long-term data are necessary, followed by successful implementation to overcome barriers such as limited equipment availability and lack of adequate surgical skills [30].

A further treatment option is the micropulse transscleral cyclophotocoagulation (MP-TSCPC); this led to an IOP reduction by 7.5 mmHg in an African population after one year, lowering glaucoma medication use from 1.5 to 1.2 substances [31]. Although limited studies are reporting within Africa, available studies in industrialized countries highlight its promise as a primary surgical option for POAG, providing modest IOP reduction with few serious complications [17].

### 2.3. Medical Therapy

Medical therapy or laser trabeculoplasty is typically the first line of treatment due to its low side effect profile in countries with available medication and laser devices [32]. In Africa, strong evidence on medical therapy and its clinical efficacy and long-term outcomes remains limited. This review identified a notable scarcity of studies evaluating commonly used agents such as prostaglandin analogues, beta-blockers, and fixed-dose combinations within the region. Among the available evidence, Timolol 0.5% proved less efficacious compared to SLT in reducing IOP, with a 31% and 61% success rate, respectively. Comparative studies also report prostaglandin analogues as being more effective in patients of African descent than in those of European descent, while beta-adrenergic receptors have been suggested to be less effective, although the evidence is inconsistent [8]. This gap in outcome-based research poses challenges for evidence-informed policy and practice, especially in diverse African healthcare settings. Compounding this issue is a high reported rate of non-adherence, exceeding 50%, driven by a range of factors including forgetfulness, complexity of dosing schedules, cultural beliefs about the ineffectiveness of eye drops, and poor self-efficacy in disease management [33]. Medication adherence is further challenged by concerns over drug quality, with issues including incorrect pH, reduced volume and concentration, and bacterial contamination [34]. Microbial contamination of topical glaucoma medications has also been reported, with contamination rates ranging from 12.9% to 28% [35,36,37]. Additionally, an assessment of eye drop instillation techniques by glaucoma patients revealed that 63% of participants demonstrated poor eye drop administration techniques [38].

## 3. Limitations and Future Implications

This review has some limitations that warrant consideration. First, only high-quality studies providing strong evidence, mainly RCTs and prospective non-randomized studies, were included, while retrospective and lower-level evidence studies were excluded. Although this strengthened the reliability of the findings, it may have omitted some unique insights from these available studies. Secondly, variations in reporting standards and outcome definitions among included studies limited direct comparability. Finally, the language restriction to English may have resulted in the exclusion of some pertinent studies. In addition, although studies from both sub-Saharan and North African regions were included, the majority of available data were derived from Black African populations, which may limit the generalizability of the findings across the diverse African continent. Furthermore, due to the limited evidence on tube shunt procedures and minimally invasive glaucoma surgery (MIGS), we were unable to adequately assess their efficacy, underscoring the need for future interventional studies in these surgical areas of glaucoma care. Also, this review focused on synthesizing evidence-based studies reporting on treatment modalities and clinical outcomes, rather than issues related to medication accessibility, affordability, or health-system implementation. As a result, important contextual barriers to glaucoma care in Africa were not addressed and remain an area for future investigation. Furthermore, evidence on fixed-dose combinations and preservative-free formulations, in most African regions remain scarce. Despite these constraints, this review offers a foundational framework of robust synthesis for the most reliable evidence on glaucoma treatment and outcomes in African populations.

Of note, improving glaucoma outcomes in Africa requires a multi-faceted approach that addresses treatment accessibility, effectiveness, sustainability, and workforce capacity. Expanding access to proven interventions, such as trabeculectomy and SLT, should be prioritized through training programs, affordable equipment provision, and scaling of surgical expertise. Evidence-based research should evaluate long-term treatment outcomes, comparative effectiveness, and cost-efficiency of newer surgical and laser techniques within African settings, while standardized definitions of treatment success can enhance clinical benchmarking and guide context-specific practice. In resource-constrained areas, combined trabeculectomy with manual small incision cataract surgery (MSICS) has shown promise, even when performed by ophthalmology fellows, achieving complete success rates of 55% and qualified success rates of 83% at 1 year [39]. Early follow-up by mid-level eye care workers using IOP as a control indicator, alongside glaucoma registers to identify defaulters, may optimize outcomes in underserved populations. Additionally, early detection facilitated by brief training of community health workers in portable diagnostic tools and free online glaucoma risk calculators can allow timely intervention with MSICS, effectively lowering IOP and mitigating disease progression [40,41]. Of note, Africa faces a severe shortage of glaucoma surgeons relative to disease burden, with only 2.5 ophthalmologists per million people in sub-Saharan Africa, where glaucoma prevalence remains high [3]. Limited surgical training capacity exacerbates the challenge; among 24 sub-Saharan countries, only 56 ophthalmology training institutions exist, with a median training duration of four years [42]. Simulation-based surgical education, as demonstrated in the GLAucoma Simulated Surgery (GLASS) trial, can significantly improve trainee competency. In this trial, 51 trainee ophthalmologists who had performed no prior trabeculectomies achieved surgical competency rates of 76.1% after intensive simulation-based training, compared to 24.4% in conventionally trained controls [43]. Scaling such innovative training programs is essential to increase the glaucoma surgical workforce and improve patient outcomes. Strengthening primary-level capacity for diagnosis and timely referral, integrating glaucoma care into national eye health strategies, and investing in health system infrastructure are vital to reducing treatment failure and preventing irreversible blindness. Coordinated efforts linking clinical research, training, community-based screening, and policy implementation are required to ensure sustainable improvements in glaucoma management across the continent. Collectively, incorporating alternative surgical approaches, community-level screening strategies, and simulation-based training programs, Africa can enhance glaucoma care, reduce treatment gaps, and prevent avoidable blindness in high-burden, resource-limited settings.

## 4. Conclusions

Glaucoma imposes a substantial and preventable burden across Africa, with treatment outcomes shaped by population-specific factors, limited resources, and workforce constraints. This review demonstrates that surgical interventions, particularly trabeculectomy augmented with anti-metabolites, achieve the most consistent IOP reduction and long-term success, though they carry higher complication risks. Alternative procedures, including deep sclerectomy, viscocanalostomy, canaloplasty, and combined trabeculectomy MSICS, offer effective and safer options, especially in resource-limited settings. Laser therapies, SLT, and transscleral cyclophotocoagulation, achieve meaningful IOP reduction with low complication rates, while medical therapy shows variable efficacy, high non-adherence, and limited outcome-based evidence in African populations. Pediatric interventions, such as goniotomy, demonstrate significant efficacy but remain underreported. Collectively, these findings underscore the urgent need for context-specific strategies that combine effective surgical and laser therapies with strengthened health systems and workforce capacity to improve glaucoma care. Implementing early detection programs and evidence-informed policies can substantially reduce treatment gaps, prevent irreversible vision loss, and mitigate the continent’s disproportionate glaucoma burden.

## Figures and Tables

**Table 1:** Summary of Glaucoma Interventions and IOP Outcomes in African Populations.

Region	Study Design	Age (±SD)	Sample Size (Patients/Eyes)	Glaucoma Type	Intervention Group	Control Group	Success Definition	Baseline IOP (mmHg)
Congo Mwanz and Kabasele [10]	RCT	-	11/22	POAG	TE with MMC	TE without MMC	IOP ≤ 21 mmHg	31/32.1
Ghana Egbert et al. [11]	RCT	59.7 (22–83)	55/-	POAG, CACG, ARG	TE with 5-FU	TE without 5-FU	IOP ≤ 20 mmHg	29.2 (18–46) /33.4 (16–76)
Kenya Yorston et al. [12]	RCT	-	68/68	POAG	TE with 5-FU	TE without 5-FU	-	33.8 ± 8.8/ 32.4 ± 9.2
South Africa Kirwan et al. [13]	RCT	-	320/-	POAG, PXG	TE with beta radiation	TE without beta radiation	Surgical failure defined as IOP > 21 mmHg	-
Ghana Singh et al. [14]	RCT	53.6	85/85	POAG	TE with 5-FU	TE with MMC	(1) IOP ≤ 21 mm Hg with or without medical therapy; (2) IOP of <15 mm Hg	31.4
Nigeria Mielke et al. [15]	RCT	-	39/39	POAG	DS with MMC	DS without MMC	IOP < 18 mmHg	29.5 ± 8.0/ 26.4 ± 5.88
Tanzania Philippin et al. [16]	RCT	66.3 ± 11.6	201/382	POAG	SLT group	Timolol Group	(1) ≤18 mm Hg for advanced; (2) ≤21 mm Hg for moderate glaucoma	26.96 ± 7.52/26.38 ± 6.28
Ghana Egbert et al. [17]	RCT	60.9 ± 12.9	92/-	POAG	Diode laser TSCPC	Glaucoma medications	20% decrease in IOP	29.0 ± 8.9
Nigeria Atima et al. [18]	RCT	-	35/66	PCG	Goniotomy (Kahook blade, microvitreoretinal blade, 23G needle group)	Groups compared against each other	-	Kahook = 30.3; MV blade = 28.9; 23G needle = 30.0
Congo Kalala et al. [19]	Prospective study	64.5 ± 14.0	34/51	POAG	Penetrating DS	-	≥20% decrease in IOP and (1) ≤12 mm Hg; (2) ≤15 mm Hg; (3) ≤18 mm Hg	Medicated: 20.2 ± 6.1 Unmedicated: 30.7 ± 9.8
South Africa Grieshaber et al. [20]	Prospective study	49.8 ± 15.7	60/60	POAG	Canaloplasty	-	-	45.0 ± 12.1
South Africa Stegmanm et al. [21]	Prospective study	-	157/214	POAG	Viscocanalostomy	-	-	47.4 ± 13
Ethiopia Soboka et al. [22]	Prospective study	57.3 ± 10.2	61/95	POAG, PXG	SLT	-	20% decrease in IOP	24.3 ± 2.5
Egypt Abdelrahman and Eltanamly [23]	Prospective study	53.2	106/106	POAG	SLT	-	IOP < 21 mmHg and 20% decrease	19.55 ± 4.8

IOP—Intraocular Pressure; RCT—Randomized Controlled Trial; TE—Trabeculectomy; 5-FU—5-Fluororacil; MMC—Mitomycin-C; TSCPC—Transscleral cyclophotocoagulation; DS—Deep sclerectomy; SLT—Selective Laser Trabeculoplasty; POAG—Primary open angle glaucoma; CACG—Chronic angle-closure glaucoma; ARG—Angle recession glaucoma; PCG—Primary congenital glaucoma; PXG—Pseudoexfoliative glaucoma.

**Table 2:** Post-Treatment IOP Outcomes, Success Rates, and Complications of Glaucoma Therapies across African populations.

Region	Intervention Therapy	Control Therapy	IOP After Therapy (mmHg ±SD)		Success Rate (%)		IOP Reduction Rate (%)		Follow-Up (Months)	Complications
			Intervention	Control	Intervention	Control	Intervention	Control		
Congo Mwanza and Kabasele [10]	TE with MMC	TE without	12.7	17.8	81.8	63.8	57.9	42.9	20	Persistent hypotonia, transitory hypotonia, superficial punctate keratitis, shallow AC
Ghana Egbert et al. [11]	TE with 5-FU	TE without 5-FU	17.3	24.5	71	32	-	-	8 to 30	Wound leak, flat anterior chamber, Epithelial defects
Kenya Yorston et al. [12]	TE with 5-FU	TE without 5-FU	16.9 ± 5.8	17.4 ± 6.1	70.6	88.8	-	-	6 to 24	Shallow AC
South Africa Kirwan et al. [13]	TE with beta radiation	TE without beta radiation	11.5	13.5	-	-	61	55	20/18	AC trauma, clinically significant uveitis, Flat AC, Hypotony, Suprachoroidal haemorrhage, blebitis, misdosage
Ghana Singh et al. [14]	TE with 5-FU	TE with MMC	16.3	13.7	73	93.2	-	-	10.0 ± 4.41	Flat AC, Encapsulated bleb, cataract, Hypotony
Nigeria Mielke et al. [15]	DS with MMC	DS without MMC	-	-	13	24	-	-	16.4 ± 11.3	Shallow AC, conjunctival edge leak, late iris incarceration, increased IOP
Tanzania Philippin et al. [16]	SLT group	Timolol Group	-	-	-	-	61	31	12	Conjunctival injection
Ghana Egbert et al. [17]	Diode laser TSCPC	Antiglaucoma medications or TE	25.7 ± 10.3	-	-	-	20% (37 eyes); 30% (24 eyes)	-	13.2 ± 6.0	Pain, mild iritis, transient conjunctival burns, hyphema, atonic pupil
Nigeria Atima et al. [18] (PCG)	Goniotomy (Kahook blade, MV blade, and 23G needle)	Groups compared against each other	Kahook blade = 9.7; MV blade = 9.5; 23-gauge needle = 8.9	-	-	-	-	-	12	-
Congo Kalala et al. [19]	Penetrating DS	-	12.1 ± 4.1	-	64.7	-	40.3% (Medicated baseline) 92.4% (Unmedicated baseline)	-	12	Bled fibrosis; iris incarceration
South Africa Grieshaber et al. [20]	Canaloplasty	-	13.3 ± 1.7	-	77.5	-	-	-	30.6 ± 8.4	Descemet’s detachment, false passage of catheter, elevated IOP, transient microhyphema
South Africa Stegmanm et al. [21]	Viscocanalostomy	-	16.85 ± 8.0	-	82.7	-	64	-	35	Hyphema, intraoperative vitreous hemorrhage
Ethiopia Soboka et al. [22]	SLT	-	17.6 ± 3.4	-	60	-	27.6	-	12	Transient ocular pain, brow ache, headache, and/or blurring of vision, anterior chamber reaction, IOP spike ≥ 6 mmHg
Egypt Abdelrahman and Eltanamly [23]	SLT	Antiglaucoma medication	16.03 ± 2.8	-	70	-	18	-	18	Mild flare and cells in AC, discomfort during procedure, increased IOP

IOP—Intraocular Pressure; TE—Trabeculectomy; 5-FU—5-Fluororacil; MMC—Mitomycin-C; TSCPC—Transscleral cyclophotocoagulation; DS—Deep sclerectomy; SLT—Selective Laser Trabeculoplasty; AC—Anterior Chamber.

## Data Availability

No new data were created or analyzed in this study.

## Abbreviations

The following abbreviations are used in this manuscript:

IOP—Intraocular Pressure; RCT—Randomized Controlled Trial; TE—Trabeculectomy; 5-FU—5-Fluororacil; MMC—Mitomycin-C; TSCPC—Transscleral cyclophotocoagulation; MP-TSCPC—Micropulse transscleral cyclophotocoagulation; DS—Deep sclerectomy; SLT—Selective Laser Trabeculoplasty; POAG—Primary open angle glaucoma; PACG—Primary angle closure glaucoma; CACG—Chronic angle-closure glaucoma; PCG—Primary congenital glaucoma; PXG—Pseudoexfoliative glaucoma; AC—Anterior Chamber, MIGS—Minimally Invasive Glaucoma Surgery; MSICS—Manual Small Incision Cataract Surgery; GLASS—GLAucoma Simulated Surgery
